# Draft Genome Sequence of *Pseudomonas* sp. Strain Ant30-3, a Psychrotolerant Bacterium with Biodegradative Attribute Isolated from Antarctica

**DOI:** 10.1128/genomeA.00522-14

**Published:** 2014-06-05

**Authors:** Hyunmin Koo, Malay K. Basu, Michael Crowley, Jackie Aislabie, Asim K. Bej

**Affiliations:** a Department of Biology, University of Alabama at Birmingham, Birmingham, Alabama, USA; b Pathology Informatics, Department of Pathology, University of Alabama at Birmingham, Birmingham, Alabama, USA; c Heflin Center for Genomic Sciences, University of Alabama at Birmingham, Birmingham, Alabama, USA; d Landcare Research, Hamilton, New Zealand

## Abstract

*Pseudomonas* sp. strain Ant30-3, isolated from fuel-contaminated Antarctic soil, exhibited distinctive psychrotolerant attributes and the potential for degrading aromatic hydrocarbon compounds at cold temperatures. We report here the 6.14-Mb draft genome of Ant30-3, which will provide insights into the genomic basis for the psychrotolerant and biodegradative properties of this bacterium.

## GENOME ANNOUNCEMENT

*Pseudomonas* sp. strain Ant30-3, isolated from fuel-contaminated soil near the former Vanda Station located in the McMurdo Dry Valleys, Antarctica, manifests growth at near subzero temperatures, as well as freeze tolerance, expression of a *capB-*encoded cold acclimation protein (1, 2), the secretion of a copious amount of extracellular polymeric substance (EPS) (3), and the antifreeze property of cellular proteins that promote the growth of stable hexagonal water crystals at subzero temperatures (4). Also, aromatic hydrocarbon-degrading genes were detected in Ant30-3 by PCR (5). Thus, Ant30-3 manifests multifaceted cold-adaptive genetic determinants with the potential for applications in the biodegradation of hydrocarbon compounds in cold ecosystems.

The genomic DNA from Ant30-3, cultured on R2A (BD, Franklin, NJ), was extracted using the Mo Bio PowerSoil DNA purification kit. The genome was sequenced on the Illumina MiSeq platform with a 2 × 250 paired-end run. A total of 4,074,199 sequences were generated per run. The adapter and low-quality sequences were examined by FastQC (http://www.bioinformatics.babraham.ac.uk/projects/fastqc/) and then trimmed by Trimmomatic (6) before assembly. The trimmed sequences were assembled *de novo* using Velvet 1.2.10 (7) with VelvetOptimiser (http://bioinformatics.net.au/software.velvetoptimiser.shtml) to determine the optimal assembly parameters. This resulted in 266 contigs using a *k*-mer length of 225, with a G+C content of 58.6%. The contig sizes are from 449 to 225,280 bp, with a mean length of 23,083 bp and an *N*_50_ of 76,193. The total draft genome length is 6,140,162 bp, with a coverage of 316×.

The annotation of the assembled genome was conducted using the Rapid Annotations using Subsystems Technology (RAST) (8). The rRNA and tRNA genes were detected by RNAmmer (9), tRNAscan-SE (10), and ARAGORN (11). The genome was shown to encode at least 91 predicted RNAs, including 1 rRNA operon, 70 tRNAs, 1 transfer-messenger RNA (tmRNA), and 19 miscellaneous RNAs. Based on the RAST results, 5,845 protein-coding genes (CDSs) were detected, of which 2,828 were classified in a known subsystem and 3,017 were classified in the unknown subsystem. Also, RAST indicated *Pseudomonas fluorescens* Q8r1-96 to be the closest neighbor to Ant30-3.

The 133 genes related to the metabolism of aromatic compounds included genes for two catechol 1,2-dioxygenases, one catechol 2,3-dioxygenase (5), three ferredoxin-related coding genes (12), two protocatechuate 3,4-dioxygenases (12), two alkanesulfonate monooxygenases (13), and a gene cluster for the degradation of phenols, cresols, and catechol (12). Six genes belonging to the cold shock family of proteins, including CspA, CspC, CspD, and CspG, chaperonin GroEL and GroES, and the RecA protein, were found on the Ant 30-3 genome. The 98 genes related to oxidative stress genes included superoxide dismutase, catalase, peroxidase, and the cytochrome *c551* peroxidase gene, Redox (*dps* gene).

The genome sequence of *Pseudomonas* sp. Ant30-3 revealed stress-responsive genes, including cold adaptation and oxidative stress, as well as biodegradative genes that will allow us to better understand the survival mechanisms and potential for the biodegradation of spilled oil in cold ecosystems.

### Nucleotide sequence accession number.

This whole-genome shotgun project has been deposited in DDBJ/ENA/GenBank under the accession no. JMCL00000000. The version described in this paper is the first version.

## References

[1] Panicker G Aislabie J Saul D Bej AK 2002 Cold tolerance of *Pseudomonas* sp. 30–3 isolated from oil-contaminated soil, Antarctica. Polar Biol. 25:5–11. 10.1007/s003000100304

[2] Panicker G Mojib N Nakatsuji T Aislabie J Bej AK 2010 Occurrence and distribution of *capB* in Antarctic microorganisms and study of its structure and regulation in the Antarctic biodegradative *Pseudomonas* sp.30-3. Extremophiles 14:171–183. 10.1007/s00792-009-0296-5 20091073

[3] Panicker G Aislabie J Bej AK 2006 Analysis of aggregative behavior of *Pseudomonas* sp. 30–3 isolated from Antarctic soil. Soil Biol. Biochem. 38:3152–3157. 10.1016/j.soilbio.2006.02.006

[4] Bej AK Mojib N 2009 Cold adaptation in Antarctic biodegradative microorganisms, p 159–175 *In* Bej AK Aislabie J Atlas RM (ed), Polar microbiology: the ecology, biodiversity and bioremediation potential of microorganisms in extremely cold environments. CRC Press, Boca Raton, FL

[5] Panicker G Mojib N Aislabie J Bej AK 2010 Detection, expression and quantitation of the biodegradative genes in Antarctic microorganisms using PCR. Antonie Van Leeuwenhoek 97:275–287. 10.1007/s10482-009-9408-6 20043207

[6] Lohse M Bolger AM Nagel A Fernie AR Lunn JE Stitt M Usadel B 2012 RobiNA: a user-friendly, integrated software solution for RNA-Seq-based transcriptomics. Nucleic Acids Res. 40:W622–W627. 10.1093/nar/gks540 22684630 PMC3394330

[7] Zerbino DR Birney E 2008 Velvet: algorithms for *de novo* short read assembly using de Bruijn graphs. Genome Res. 18:821–829. 10.1101/gr.074492.107 18349386 PMC2336801

[8] Aziz RK Bartels D Best AA DeJongh M Disz T Edwards RA Formsma K Gerdes S Glass EM Kubal M Meyer F Olsen GJ Olson R Osterman AL Overbeek RA McNeil LK Paarmann D Paczian T Parrello B Pusch GD Reich C Stevens R Vassieva O Vonstein V Wilke A Zagnitko O 2008 The RAST server: Rapid Annotations using Subsystems Technology. BMC Genomics 9:75. 10.1186/1471-2164-9-75 18261238 PMC2265698

[9] Lagesen K Hallin P Rødland EA Staerfeldt HH Rognes T Ussery DW 2007 RNAmmer: consistent and rapid annotation of ribosomal RNA genes. Nucleic Acids Res. 35:3100–3108. 10.1093/nar/gkm160 17452365 PMC1888812

[10] Lowe TM Eddy SR 1997 tRNAscan-SE: a program for improved detection of transfer RNA genes in genomic sequence. Nucleic Acids Res. 25:955–964. 10.1093/nar/25.5.0955 9023104 PMC146525

[11] Laslett D Canback B 2004 ARAGORN, a program to detect tRNA genes and tmRNA genes in nucleotide sequences. Nucleic Acids Res. 32:11–16. 10.1093/nar/gkh152 14704338 PMC373265

[12] Fuchs G Boll M Heider J 2011 Microbial degradation of aromatic compounds—from one strategy to four. Nat. Rev. Microbiol. 9:803–816. 10.1038/nrmicro2652 21963803

[13] Eichhorn E van der Ploeg JR Leisinger T 1999 Characterization of a two-component alkanesulfonate monooxygenase from *Escherichia coli*. J. Biol. Chem. 274:26639–26646. 10.1074/jbc.274.38.26639 10480865

